# Unraveling the complex interplay between obesity and vitamin D metabolism

**DOI:** 10.1038/s41598-024-58154-z

**Published:** 2024-03-30

**Authors:** Bashar Alzohily, Asma AlMenhali, Salah Gariballa, Nayla Munawar, Javed Yasin, Iltaf Shah

**Affiliations:** 1 Department of Chemistry, College of Science, UAE University, 15551, Al Ain, UAE; 2 Department of Biology, College of Science, UAE University, 15551, Al Ain, UAE; 3 Internal Medicine, Faculty of Medicine and Health Sciences, UAE University, 15551, Al Ain, UAE

**Keywords:** Vitamin D, Vitamin D metabolites, Epimers of vitamin D, UHPLC-MS/MS, Vitamin D blood measurement test, Obese subjects vitamin D deficiency, Biological techniques, Biomarkers, Health care, Chemistry

## Abstract

Vitamin D deficiency and obesity are a worldwide health issue. Obesity refers to the accumulation of excessive fats in the body which could lead to the development of diseases. Obese people have low vitamin D levels for several reasons including larger volume of distribution, vitamin D tightly bound in fatty tissues, reduced absorption, and diets with low vitamin D. Accurately measuring vitamin D metabolites is challenging. The Ultra-High-Performance Liquid Chromatography-Tandem Mass Spectrometry (UHPLC-MS/MS) method was developed and validated for the analysis of vitamin D metabolites in the serum. Blood samples were collected from 452 subjects which consisted of baseline (vitamin D deficient obese subjects), follow-up (supplemented obese subjects), and healthy volunteers. The vitamin D metabolites were separated adequately by the developed UHPLC-MS/MS method. Moreover, the validation criteria for the method were within an acceptable range. The baseline, follow-up and even healthy volunteers were deficient in 25OHD3 and 25OHD2. The baseline and healthy subjects had comparable concentration of vitamin D2 and D3. However, healthy subjects had a higher concentration of 25OHD and its epimer compared to the baseline subjects. The vitamin D3 was increased significantly in the follow- up subjects; therefore, the 25OHD3 was increased significantly compared to the baseline as well; however, the increase was insufficient to achieve the optimal range. The UHPLC-MS/MS method test was applied successfully on estimation of vitamin D metabolites in subjects. This study indicates the significance of taking into account the metabolic and storage effects when evaluating the vitamin D status in obese subjects.

## Introduction

Obesity is a worldwide health problem; it refers to the excessive amount of fats accumulated in the body, which may be detrimental to human health^1^. Obesity could lead to the development of several diseases, such as cardiovascular disease, high blood pressure, cancer, and diabetes; subsequently, it will raise morbidity and mortality rates. Additionally, it could result in social discrimination and reduced physical fitness^2,3^. Obesity can be related to lifestyle or eating habits and genetic factors^4,5^. Numerous studies have confirmed a correlation between obesity and vitamin D deficiency; however, the causal association has not been fully understood^6^. The dilution of vitamin D in a large body volume could be a potential reason behind the inverse association between vitamin D plasma levels and Body Mass Index (BMI). Although obese and lean individuals have similar amounts of vitamin D, the concentration of vitamin D seems to be higher in lean compared to obese individuals, owing to its dilution in a massive volume of obese individuals^7^. Nevertheless, if volumetric dilution reduces 25-hydroxyvitamin-D (25OHD) amounts in obese individuals, weight loss could help in increasing 25OHD levels in serum; however, inconsistent findings have been demonstrated by weight loss study. For example, Mason et al.^8^ noted that when overweight and obese postmenopausal subjects followed a diet and workout program for weight loss, they found that 25OHD serum levels didn’t increase significantly. In other words, when the volunteers have lost < 5%, 5–9.9%, 10–14.9%, and > 15% of their weight, the 25OHD levels raised by 2.1, 2.7, 3.3, and 7.7 ng/ml^8^. One research reported that 25OHD concentration is increased more in lean individuals compared to obese individuals upon vitamin D supplementation^9^. According to Drincic et al.^10^, vitamin D supplementation amounts in obese individuals must be reconsidered to reduce the 25OHD concentration differences between obese and lean individuals. Another research noted that a crucial hormonal pathway for the health of the skeleton may be inhibited when the body has a huge amount of fats. For example, a leptin hormone from fat cells could inhibit the synthesis pathways of vitamin D active metabolites^11^. When adipose tissue sequestrates vitamin D3, this will probably lead to reduce bioavailability of D3 from skin and dietary sources especially in obese individuals since they have high mass of fat cells; therefore, vitamin D3 serum levels will be insufficient or deficient in obese subjects^12,13^. Some research mentioned that 1-α-25 dihydroxy vitamin-D (1α,25(OH)_2_D) could intercede in adipogenesis inhibition via actions controlled by vitamin D- dependent receptors; from this view, large number of preadipocytes could be differentiated when vitamin D is deficient or insufficient^1,14^. Other studies reported that the increase in the 25OHD serum concentration was 53% less in obese subjects compared to healthy body weight volunteers after sunlight exposure. This suggests that, despite sunlight exposure, obese individuals experienced a smaller increase in vitamin D levels in their blood compared to those with a healthy body weight^1,13^. Multiple research investigations have indicated that vitamin D deficiency can contribute to higher body fat levels. This is thought to occur via elevated levels of parathyroid hormone and greater calcium entry into adipocytes, ultimately enhancing the process of lipogenesis^1^.

Vitamin D is a lipid-soluble steroid that plays a crucial role in calcium and phosphorous homeostasis, as well as good maintenance and development of the skeleton^15^. The body's exposure to artificial ultraviolet-B (UV-B) or sunlight is considered a major source of vitamin D; also, vitamin D can be obtained from dietary sources like fish, eggs, milk, cereals, and vitamin D supplements^16^. Deficiency in vitamin D may lead to a number of diseases, including rickets, Parkinson’s, arthritis, growth retardation, cardiovascular risk, Alzheimer’s, and dementia^16,17^.

**Figure 1:**
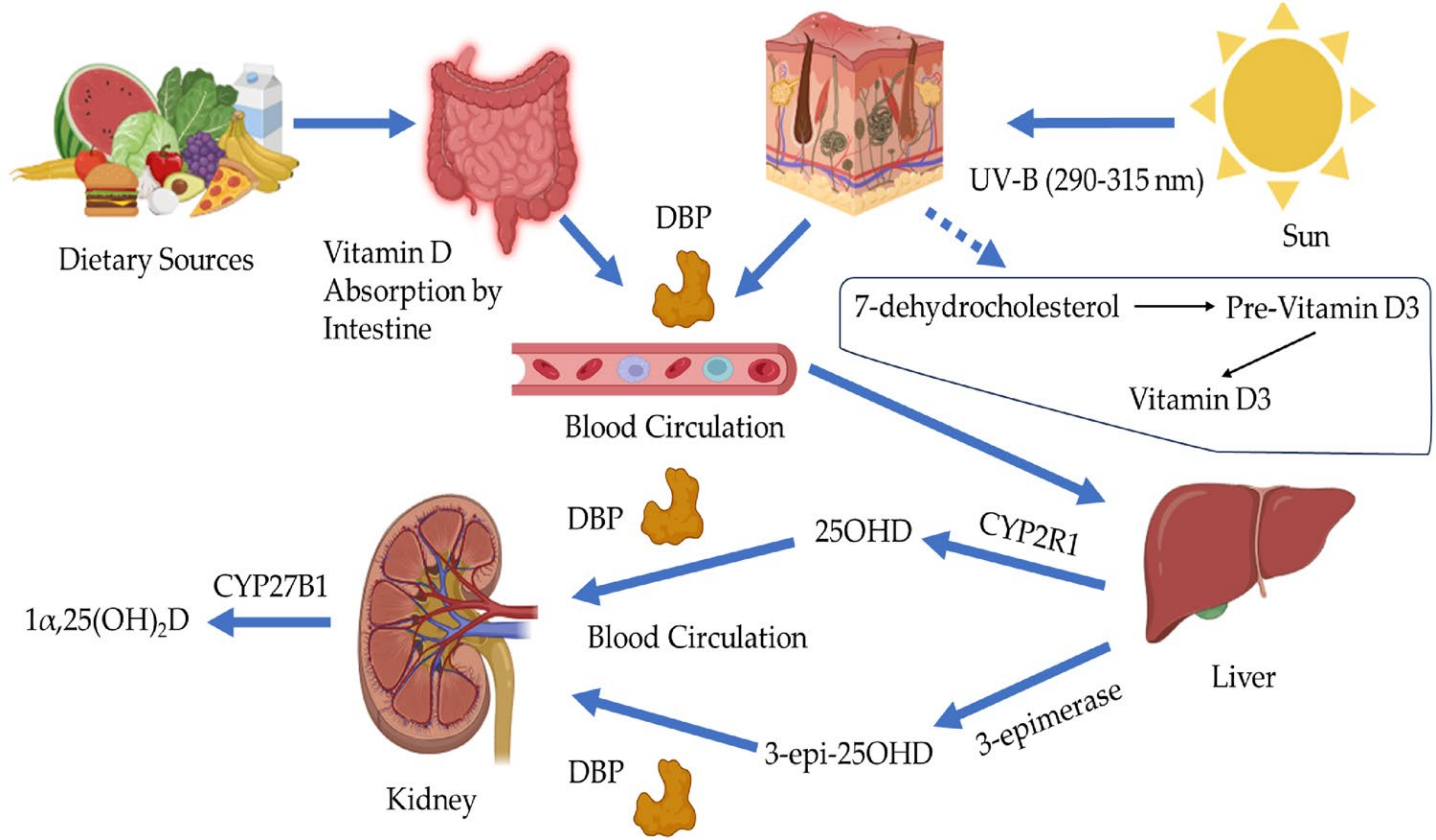
Vitamin D Metabolism. The sunshine activates the conversion of 7-dehydrocholesterol to pre-vitamin D3 in which undergoes thermal isomerization to yield vitamin D3. Vitamin D from dietary sources is absorped by intestine. Vitamin D in the skin and intestine binds with DBP which will be transported through blood toward the liver. The CYP2R1 enzyme hydroxylates the vitamin D to form 25OHD in the liver; also, 25OHD could be epimerized by 3-epimerase enzyme to form 3-epi-25OHD. After that, the 25OHD and its epimer will bind with DBP to be transported to the Kidney where the active form of vitamin D (1α,25(OH)_2_D) will be produced by CYP27B1.

There are two categories of vitamin D: The first one is from animal origin, which is vitamin D3 (cholecalciferol), and the second one is from plant origin, which is vitamin D2 (ergocalciferol). Vitamin D3 is synthesized from 7-dehydrocholesterol, a precursor to vitamin D3. Vitamin D2 is created from ergosterol, a precursor to vitamin D2^15^. Under the ultraviolet B light (medium wavelength, 290–315 nm), the 7-dehydrocholesterol is converted endogenously in the skin to vitamin D3. The intestine absorbs vitamin D from food sources and encapsulates it into chylomicrons to be released into the bloodstream. After that, vitamin D Binding Protein (DBP) carries the vitamin D to the liver, where the vitamin D will be converted to 25-hydroxyvitamin-D (25OHD) metabolite with the help of 25-hydroxylase (CYP2R1) enzyme. Also, 25OHD could be epimerized to 3-epi-25OHD by 3-epimerase enzyme^15,16^. Following that, 1α-hydroxylase (CYP27B1) enzyme will convert 25OHD to 1-α-25 dihydroxyvitamin-D (1α,25(OH)_2_D) in the kidney, as shown in Fig. 1^15,16^.

Some studies demonstrate that 7-dehydrocholesterol and vitamin D3 can undergo an alternative pathway since they act as a good substrates for CYP11A1 enzyme^18,19^. CYP11A1 can catalyze the sequential hydroxylation of 7-dehydrocholesterol followed by side chain cleavage to form 7-dehydropregnenolone (7DHP): 7DHC → 22(OH)7DHC → 20,22(OH)_2_7DHC → 7DHP. On the contrary, CYP11A1 involves successive hydroxylations in the vitamin D metabolism that start primarily at C20 or C22 without leading to the side chain cleavage : D3 → 20(OH)D3 + 22(OH)D3 → (OH)_n_D3. CYP24A1 and CYP27A1 can hydroxylate 20(OH)D3 at C24 or C25 and at C25 or C26 respectively. The products of the reactions mentioned above can undergo further hydroxylation at C1α by CYP27B1, with the exception of 17,20,23(OH)_3_D3, which is the last product resulting from the action of CYP11A1 on D3^18,19^.

Immunoassay is a commonly used method in clinical labs for assessing vitamin D levels in serum. However, this technique has a low specificity due to its inability to differentiate between D2 and D3; furthermore, it evaluates total 1α,25(OH)_2_D, and 25OHD concentrations^20^. US Food and Drug Administration (FDA) specifies that 25OHD3 and 25OHD2 should both be detected in 25OHD assays which is challenging to immunoassay technique because vitamin D binding protein binds to both 25OHD2 and 25OHD3, and producing antibodies to such small molecules is difficult^21^. High-Performance Liquid Chromatography (HPLC) is a common technique for measuring vitamin D^22,23^. The HPLC has solved the immunoassay limitations by separating 25OHD3 and 25OHD2, making the HPLC technique more accurate and precise. However, this technique has flaws, including its low sensitivity and need for a significant sample volume^24^.

Liquid Chromatography-Tandem Mass Spectrometry (LC–MS/MS) is the best technique for accuracy, precision, specificity, sensitivity, and sample volume. For instance, this technique solves all the limitations of immunoassay and HPLC methods by separating compounds based on their polarities, molecular weights, and mass-to-charge (m/z) ratios. Moreover, it has a very low Limit of Detection (LOD) and needs a small sample volume^25^.

Epimers are isomeric compounds that share the same chemical formula and differ in stereochemical configurations at a specific atom. The epimers of vitamin D have different orientations of the hydroxyl group at the C-3 position^26^. 3-epi-25-hydroxyvitamin-D3 (3-epi-25OHD3) is a well-known epimer. Separating vitamin D from its epimer is essential to avoid overestimating vitamin D measurements and get accurate results. Other two compounds act as an isobar and could interfere with the actual measurement of 25OHD3; the first one is 7-α-hydroxy-4-cholesten-3-one (7αC4, endogenous bile acid precursor), and the second one is 1α-hydroxyvitamin D3(1αOHD3, exogenous pharmaceutical compound)^27^.

The aim of this study is to provide quantitative and qualitative information for vitamin D assessment in the blood of the obese subjects and to assess whether the vitamin D supplements could raise the active forms of vitamin D metabolites or their epimer or both.

## Results and discussion

### Ultra-high-performance liquid chromatography-tandem mass spectrometry assay

**Figure 2:**
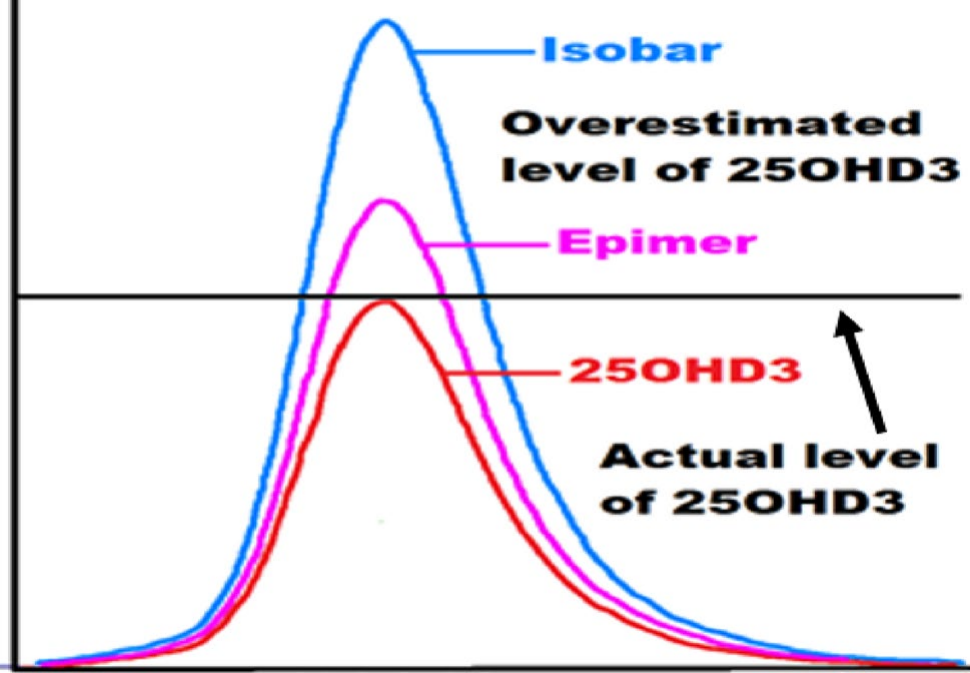
Overestimation of 25OHD3 levels. The epimer and isobar of 25OHD3 overestimate the 25OHD3 levels when using mass spectrometry technique.

**Figure 3:**
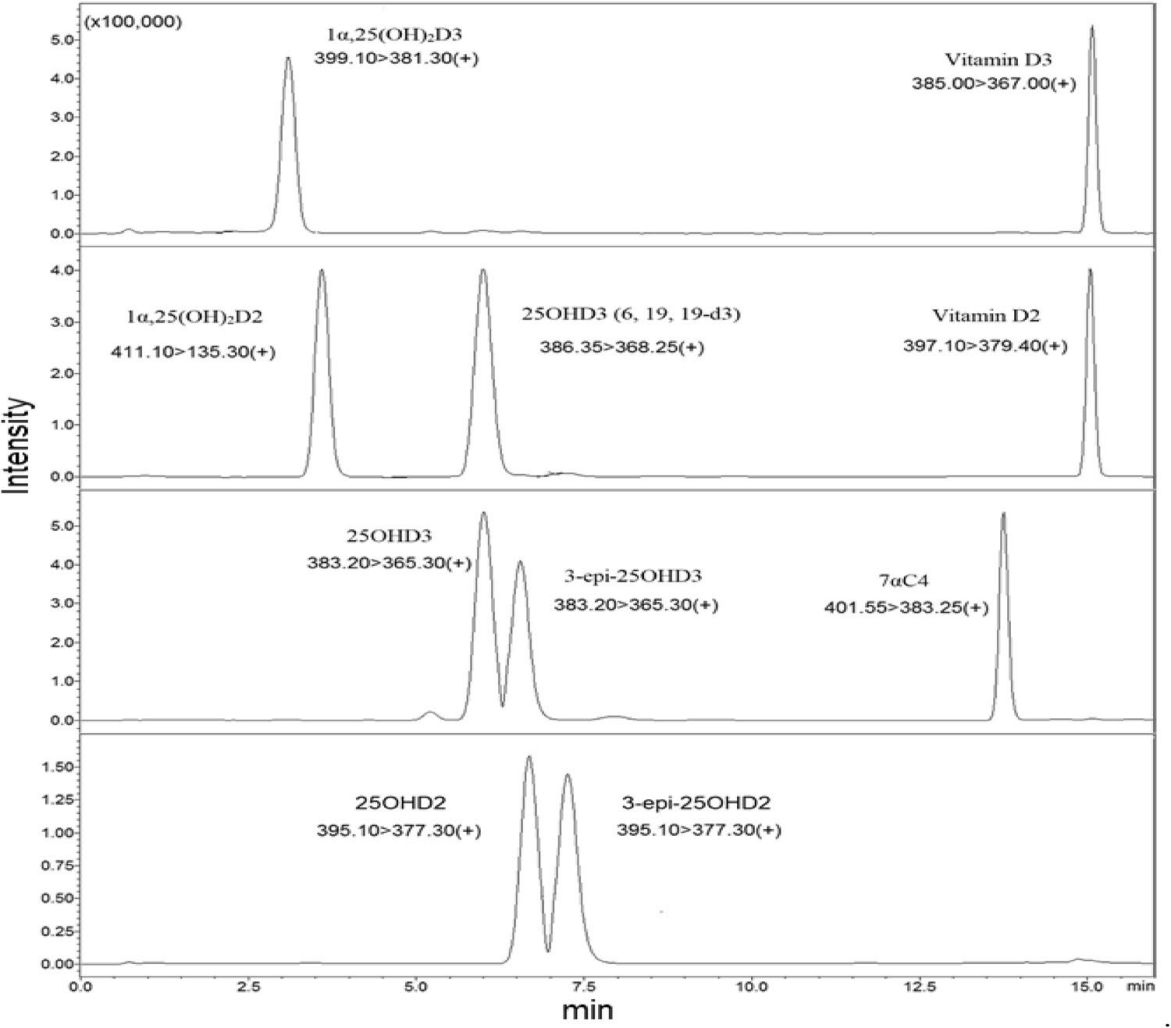
Vitamin D metabolites Chromatogram.The 25OHD3 and its isobar and epimer were adequately separated based on their polarities and retention times. Also, Other vitamin D metabolites were separated from each other sufficiently.

Using ultra-high-performance liquid chromatography (UHPLC) system, the vitamin D metabolites and their epimers (3-epi-25OHD) and isobar (7αC4) were separated adequately based on their retention times and polarities, as shown in Fig. 3. Figure 3 illustrates the chromatogram of standard vitamin D metabolites prepared in a surrogate matrix (artificial serum), the vitamin D metabolites chromatogram in human serum samples is provided in the Electronic Supplementary Material (ESM). Some vitamin D metabolites have similar retention times but different masses, as shown in Table 1; however, it will not be a problem since the tandem mass spectrometry (MS/MS) system can distinguish between these metabolites based on their masses, which makes the UHPLC-MS/MS technique powerful. Moreover, vitamin D metabolites and their epimers have similar structures, but their retention times are different, as shown in Table 1. 25OHD3 and its epimer and isobar have the same masses; therefore, analyzing these metabolites using only mass spectrometry will provide false-positive results. For instance, since the epimer and isobar of 25OHD3 share the same masses, they will interfere and coelute during 25OHD3 analysis; therefore, it will overestimate the actual 25OHD3 levels, as shown in Fig. 2.

**Table 1:** The names, structures, and masses of vitamin D metabolites with their retention times.

Name	Vitamer structure	Mass (g/mol)	Retention time (min)
Cholecalciferol (Vitamin D3)		384.64	15.104
Ergocalciferol (Vitamin D2)		396.65	15.072
25-Hydroxyvitamin D3 (25OHD3)		*400.64* *382.62* (-H_2_O)	6.008
*3-epi-25-Hydroxyvitamin D3* *(3-epi-25OHD3)*		*400.64* *382.62* (-H_2_O)	6.552
*25-Hydroxyvitamin D2* *(25OHD2)*		*412.65* *394.63* (-H_2_O)	6.682
*3-epi-25-Hydroxyvitamin D2* *(3-epi-25OHD2)*		*412.65* *394.63* (-H_2_O)	7.257
*1α,25-Dihydroxyvitamin D3* (1α,25(OH)_2_D3)		*416.64* *398.62* (-H_2_O)	3.108
*1α,25-Dihydroxyvitamin D2* *(1α,25(OH)*_*2*_*D2)*		*428.65* *410.63* *(-H*_*2*_*O)*	3.452
*7α-Hydroxy-4-cholesten-3-one* *(7αC4)*		*400.64*	13.763
*25-Hydroxyvitamin D*_*3*_* (6,19,19-d*_*3*_*) [Internal Standard]*		*403.66* *385.64* *(-H*_*2*_*O)*	5.983

Under the applied electrospray ionization (ESI) source, the Multiple Reaction Monitoring (MRM) transitions for all vitamin D metabolites produce the protonated vitamin D compounds, [M + H]^+^. The precursor ions of some vitamin D metabolites were found to be the most sensitive after the loss of the water compound, and MRM mode was utilized to track the most sensitive product ions. The direct infusion of each vitamin D metabolite standard solution were used to optimize the MRM. The MRM transitions of vitamin D metabolites along with their collision energies are shown in Table 2.

**Table 2:** The names of the analytes, masses, precursor, and product ions along with their collision energies. Some vitamin D metabolites have the same MRM and collision energies.

No	Analyte	Mass (g/mol)	Precursor (m/z)	Product (m/z)	Collision Energy (eV)
1	Vitamin D3	384.64	385	367	− 13
			385	259	− 16
			385	91	− 55
2	Vitamin D2	396.65	397.1	379.4	− 17
			397.1	69	− 29
3	25OHD3	400.64 382.62 (–H_2_O)	383.2	365.3	− 15
			383.2	107.1	− 30
4	3-epi-25OHD3	400.64 382.62 (–H_2_O)	383.2	365.3	− 15
			383.2	107.1	− 30
5	25OHD2	412.65 394.63 (–H_2_O)	395.1	377.3	− 17
			395.1	81.1	− 38
6	3-epi-25OHD2	412.65 394.63 (–H_2_O)	395.1	377.3	− 17
			395.1	81.1	− 38
7	1α,25(OH)_2_D3	416.64 398.62 (–H_2_O)	399.1	381.3	− 14
8	1α,25(OH)_2_D2	428.65 410.63 (–H_2_O)	411.1	135.3	− 13
			411.1	133.1	− 12
9	7αC4	400.64	401.55	383.25	− 16
			401.55	97.1	− 29
			401.55	177.2	− 23
10	25OHD3(6,19,19-d3), Internal Standard	403.66 385.64 (–H_2_O)	386.35	368.25	− 15
			386.35	257.2	− 183
			386.35	95.2	− 35

The limitations of mass spectrometry and immunoassay techniques were solved when 25OHD2 and 25OHD3 and their epimers were separated and distinguished from each other by the UHPLC-MS/MS technique. Although 3-epi-25OHD3 and 25OHD2 have almost identical retention times, they exhibit different MRM and fragmentation behaviors, as shown in Fig. 3 and Table 2. Lastly, when the UHPLC and MS/MS work together, they provide a powerful combination for bioanalysis.

### Validation of the method

#### Linearity of the calibration curves and quality controls

The coefficient of determination (R^2^) was used to express the linearity of a calibration curve. The linear range for some vitamin D metabolites was 0.5 to 100 ng/ml; for 1α,25(OH)_2_D was 0.015 to 1 ng/ml, as shown in Table 3. The ratio of vitamin D metabolite's peak area to the internal standard's peak area was plotted versus the vitamin D metabolite concentration to build the calibration curve. The R^2^ for all vitamin D metabolites ranged from 0.993 to 0.999, as shown in Table 3. Three levels of Quality Controls (QCs), including Quality Control Low (QCL), Quality Control Medium (QCM), and Quality Control High (QCH) were used to test the calibration curve. The concentrations of these QCs are shown in Table 3.

**Table 3:** The values of method validation parameters for each quality control level.

No	Vitamin D metabolites	Conc (ng/ml)	Intra-day		Inter-day		% Recovery	LOD (ng/ml)	LOQ (ng/ml)	Linearity (R^2^)	Linear Range (ng/ml)
			Precision (%CV)	% Accuracy (%PE)	Precision (%CV)	% Accuracy (%PE)					
1	Vitamin D3	QCH 80	1.31	98.9	2.89	90.2	77	0.05	0.5	0.998	0.5–100
		QCM 40	2.86	99.4	2.33	89.4	75				
		QCL 20	4.13	87.1	5.32	89.8	98				
2	Vitamin D2	QCH 80	9.4	89.4	9.6	89.3	88	0.05	0.5	0.997	0.5–100
		QCM 40	11.3	97.5	13.3	94.8	89.3				
		QCL 20	6.3	99.2	11.3	93.2	105				
3	25OHD3	QCH 80	3.5	99.1	2.9	99.2	86	0.025	0.5	0.998	0.5–100
		QCM 40	2.7	97.3	3.7	99.8	89				
		QCL 20	6.1	99.9	7.4	98.1	111				
4	3-epi-25OHD3	QCH 80	3.3	89.1	8.9	99.9	80	0.05	0.5	0.998	0.5–100
		QCM 40	3.1	87.3	10.9	87.6	96				
		QCL 20	3.5	89.2	4.3	98.5	86				
5	25OHD2	QCH 80	3.3	88.7	3.8	89.3	82	0.025	0.5	0.998	0.5–100
		QCM 40	2.7	99.9	3.9	98.9	99				
		QCL 20	2.8	99.8	3.4	101.2	115				
6	3-epi-25OHD2	QCH 80	3.2	89.9	2.2	88.3	86.5	0.05	0.5	0.998	0.5–100
		QCM 40	4.9	84.6	2.7	87.1	97				
		QCL 20	7.1	85.8	3.5	88.5	89				
7	1α,25(OH)_2_D3	QCH 0.8	3.6	99.7	2.6	88.9	88	0.01	0.015	0.997	0.015–1
		QCM 0.4	4.2	86.4	4.4	87.3	82				
		QCL 0.2	4.4	89.2	5.6	88.5	97				
8	1α,25(OH)_2_D2	QCH 0.8	3.9	99.9	4.3	87.8	86	0.01	0.015	0.993	0.015–1
		QCM 0.4	4.4	99.4	6.3	98.9	87				
		QCL 0.2	3.2	98.9	5.2	89.8	89.1				
9	7αC4	QCH 80	4.1	79.9	3.1	88.1	92.45	0.1	0.5	0.999	0.5–100
		QCM 40	2.3	88.6	3.1	85.3	97				
		QCL 20	3.2	98.3	5.4	86.2	92				

#### Precision and accuracy

Precision was expressed as the coefficient of variation percentage (CV%), while accuracy was expressed as the error percentage (PE%) between the value being measured and the nominal value. Six replicates of QCs at each level (QCL, QCM, QCH) were analysed on three separate days to determine inter-assay precision and accuracy, while the QCs analysed on one day were used to evaluate intra-assay precision and accuracy. The measured values of six replicates were used to determine the standard deviation and the mean, which were utilized to evaluate the intra- and inter-day precision and accuracy, as shown in the equations below. The values of precision and accuracy are illustrated in Table 3.

1 $$ \% {\text{CV }} = \frac{{\left( {\text{Standard deviation}} \right)}}{{\left( {{\text{mean}}} \right)}} \times 100 $$

2 $$ \% {\text{PE }} = \frac{{\left( {\text{mean value}} \right)}}{{\left( {\text{nominal value}} \right)}} \times 100 $$

Generally, the PE% was ranged from 85 to 101% while CV% was less than 15%; therefore, the values of accuracy and precision were found within an acceptable range of ≤  ± 15%.

#### Recovery percentage

The recovery percentage is the amount of analyte recovered or retrieved after extraction. Two sets of three levels of QCs at six replicates of each QC level were prepared to assess extraction recovery. In the first set, the vitamin D metabolites were spiked in methanol, while in the second set, they were spiked in a surrogate matrix and the extraction method was applied on the second set. After that, the results of the analysed two sets were compared using the peak’s area in the chromatogram. The recovery percentage was determined as follows:

3 $$ \% {\text{Recovery }} = \frac{{\left( {\text{mean extracted QC values}} \right)}}{{\left( {\text{mean unextracted QC values}} \right)}} \times 100 $$

The recovery percentage of vitamin D metabolites after liquid–liquid extraction was good and it ranged from 75 to 115%. The details are shown in Table 3.

#### Sensitivity

Sensitivity is referred to as the lowest concentration of analyte that can be detected. The Limit of Quantification (LOQ) and the Limit of Detection (LOD) were used to determine the sensitivity. The LOD was assessed by diluting the analyte concentration until the Signal to Noise ratio (S/N) equals 3, while for LOQ, the S/N ratio equals at least 10. The LOD ranged from 0.01 to 0.1 ng/ml, while LOQ ranged from 0.015 to 0.5 ng/ml. The values of LOD and LOQ are shown in Table 3. LOQ and LOD values were reported by different studies. For example, Mϋller et al.^28^ mentioned the value of LOQ, which was 0.1 and 1.0 ng/ml for 25OHD3 and its epimer, respectively. Furthermore, according to Ronda et al.^29^ who used LC–MS/MS 6490, the LOQ value was 1.36 and 1.52 ng/ml while the LOD was 0.68 and 0.76 ng/ml for 25OHD3 and its epimer, respectively. Additionaly, when they used LC–MS/MS 6410, the LOQ was 1.4 ng/ml and the LOD was 0.8 ng/ml for both 25OHD3 and its epimer. In another research, the LOQ value was reported as 0.25 and 0.3 ng/ml while LOD was 0.075 and 0.09 ng/ml for 25OHD3 and its epimer, respectively^30^. In this study, the LOD was 0.025 and 0.05 ng/ml for 25OHD3 and its epimer, respectively, while LOQ was 0.5 ng/ml for both metabolites. Generally, this explain that our method is very sensitive compared to the previous studies.

#### Specificity and stability

**Figure 4:**
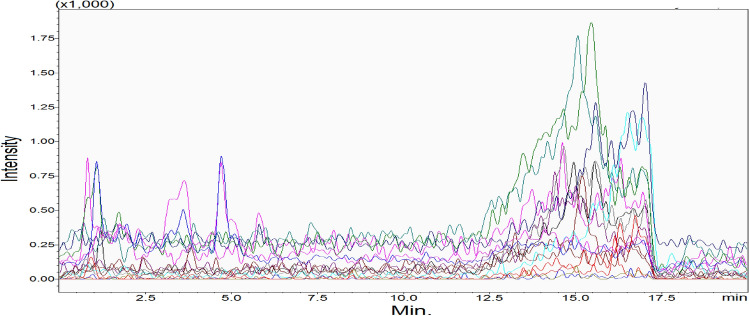
Overlaid of blank serum chromatograms illustrating the assay specificity. There were no co-eluting or interfering peaks at the same retention time of vitamin D metabolites.

**Figure 5:**
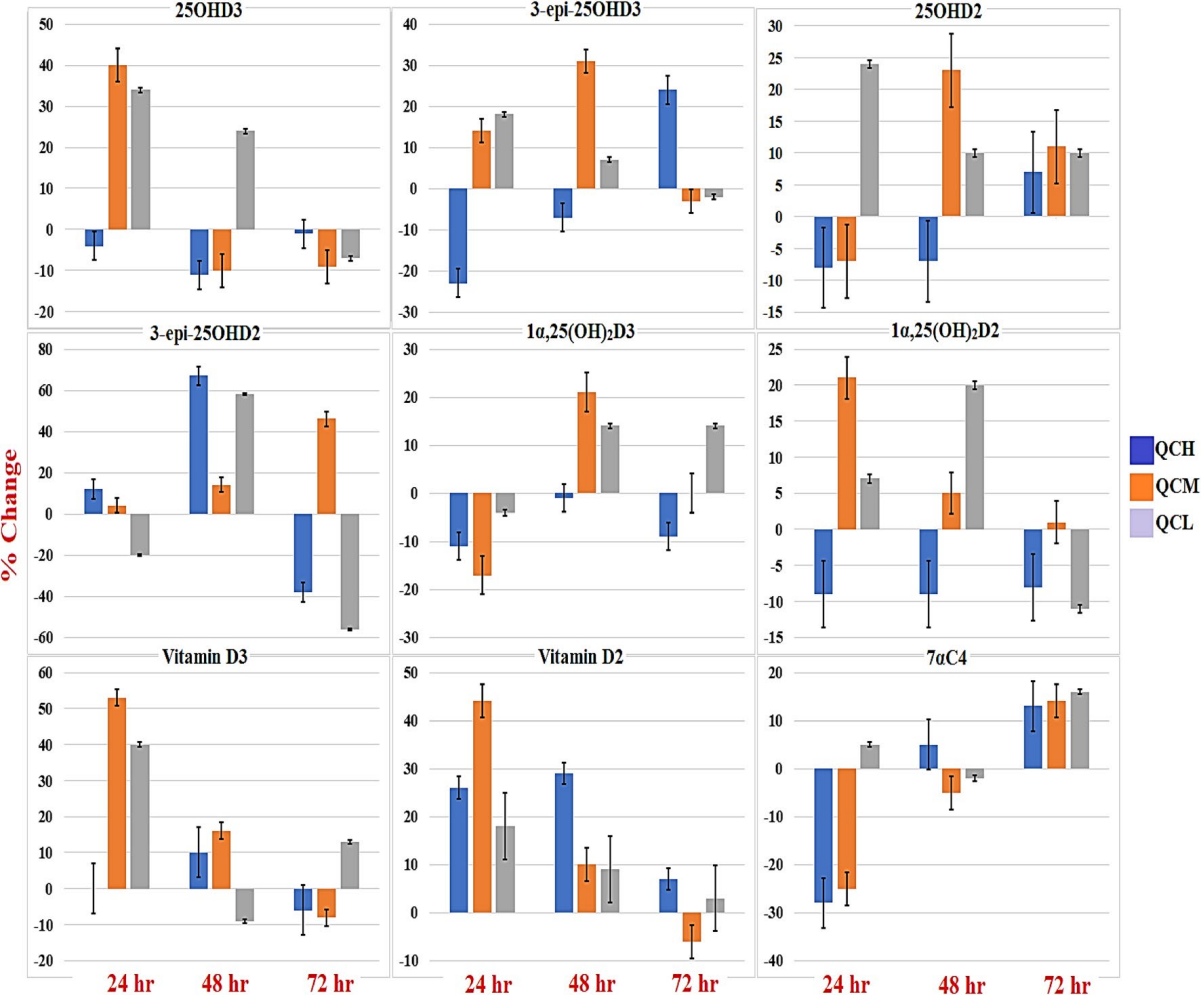
The stability (%change) of vitamin D metabolites over three cycles of freezing and thawing. The Quality Control Low (QCL), Quality Control Medium (QCM), and Quality Control High (QCH) are denoted by grey, orange, and blue bars over three intervals, respectively. The %change of every metabolite was estimated by analyzing six replicates of quality controls at each concentration level (QCL, QCM, and QCH) over three intervals of freezing and thawing. Generally, the %change fluctuated within ± 60%.

The specificity was determined by analyzing six blank serum samples (surrogate matrix). After that, the chromatograms of blank samples were overlaid to check for interfering or coeluting peaks. Figure 4 is showing the chromatogram of blank serum to confirm that there is no interfering or co-eluting peaks at the corresponding retention time of vitamin D metabolites ( the retention time of each vitamin D metabolite is mentioned in Table 1 and Fig. 3). Stability (%Change) is defined as the intactness of analyte amounts under certain storage and usage conditions over time compared to the initial amount. To evaluate the %Change, six replicates of spiked QCs at each level (QCL, QCM, QCH) were analyzed over three consecutive days when they were frozen and thawed three times at intervals of 0 h, 24 h, 48 h, and 72 h. The results of QCs at each interval were compared from time zero to calculate the %Change of vitamin D metabolites. The results illustrated that the %Change fluctuated within ± 60%, as shown in Fig. 5; therefore, the vitamin D metabolites are sensitive and less stable when they were exposed to freezing and thawing cycles. Finally, it is better to analyse fresh samples.

### Analysis of human serum samples (baseline, follow-up and healthy)

The most common vitamin D metabolite used to evaluate vitamin D status in blood samples is 25OHD. In this research, 25OHD3 and 25OHD2 concentrations were measured to diagnose vitamin D deficiency in the blood. For some reasons, measuring 1α,25(OH)_2_D metabolites is more difficult than measuring 25OHD. The first reason is that the 1α,25(OH)_2_D has a short half-life (4–15 h) compared to 25OHD (21–30 days), and the second reason is that it is less stable and exists in a low concentration in the blood^31,32^. The reference ranges of 25OHD were suggested by multiple organizations. For example, the Institute of Medicine proposed that the normal level of 25OHD is 20 ng/ml while the Vitamin D Council suggested the normal range of 25OHD is from 40 to 80 ng/ml^33,34^. One of the studies reported the reference range of a total 25OHD and 1α,25(OH)_2_D in the bloodstream. For instance, the normal levels of total 25OHD range from 25 to 80 ng/ml and the normal levels of 1α,25(OH)_2_D range from 18 to 64 pg/ml for males and 18 to 78 pg/ml for females^35^. Several studies have published various guidelines about the normal levels of 25OHD for good health; so, it appears that a consensus on a definitive adequate level of vitamin D has not been achieved^12,36–40^, as shown in Table 4.

**Table 4:** The normal range of 25OHD in serum proposed by several studies.

Study	Deficient (ng/ml)	Insufficient (ng/ml)	normal (ng/ml)	Excess (ng/ml)
^36^	< 10	10–20	20–30	> 200
^40^	< 12	12–20	20–50	> 50
^37^	< 20	21–29	30–100	> 100
^38^	< 10	10–30	30–80	> 100
^12^	< 20	21–29	30–60	> 150
^39^	< 10	10–30	30–100	> 100

**Figure 6:**
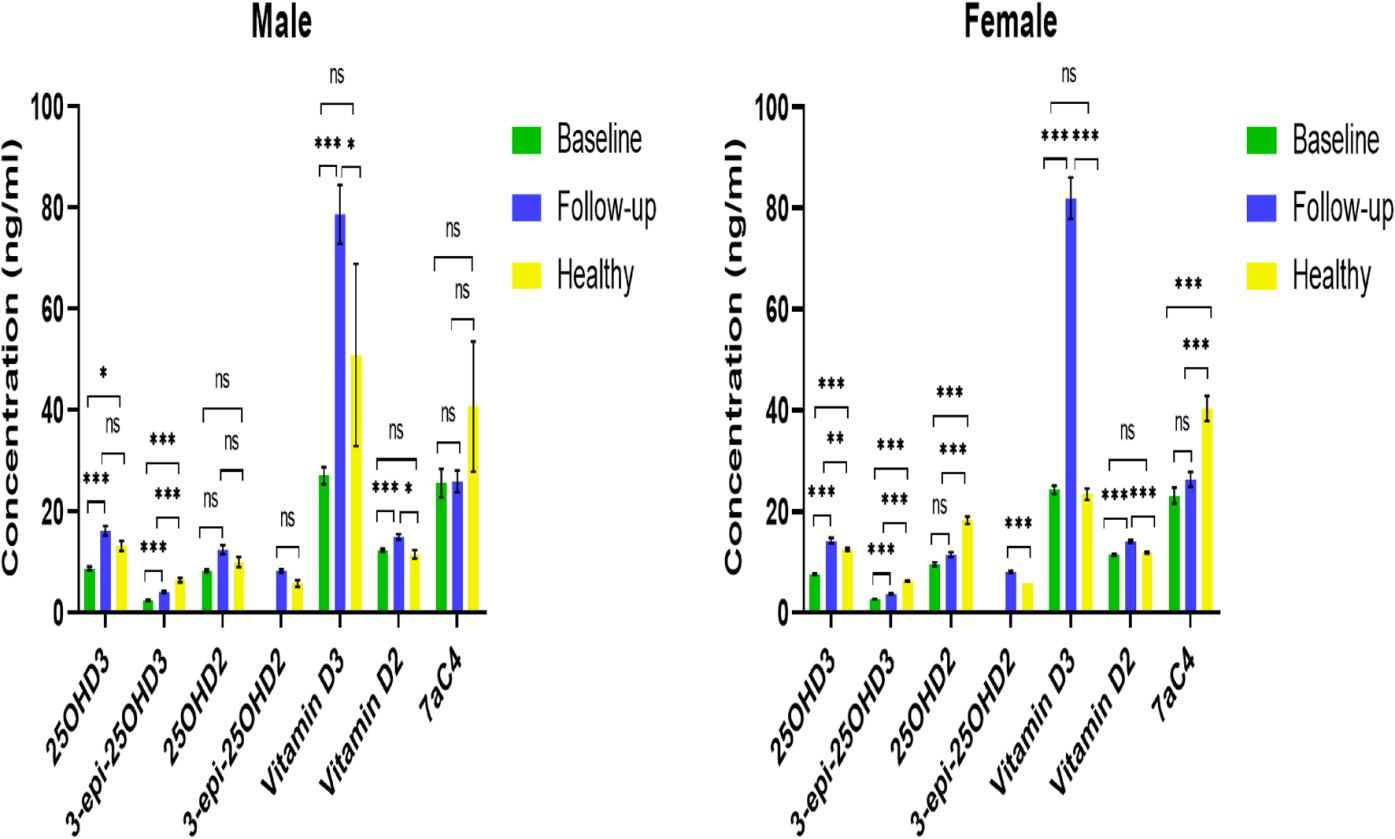
a comparison of the levels of vitamin D metabolites in plasma samples from male and female volunteers. In baseline subjects, 3-epi-25OHD2 was not detected, and in healthy, follow-up, and baseline subjects, 1α,25(OH)_2_D was not detected by the instrument. *p* < 0.05 = *, *p* < 0.01 = **, *p* < 0.001 = ***, ns = no significance.

**Figure 7:**
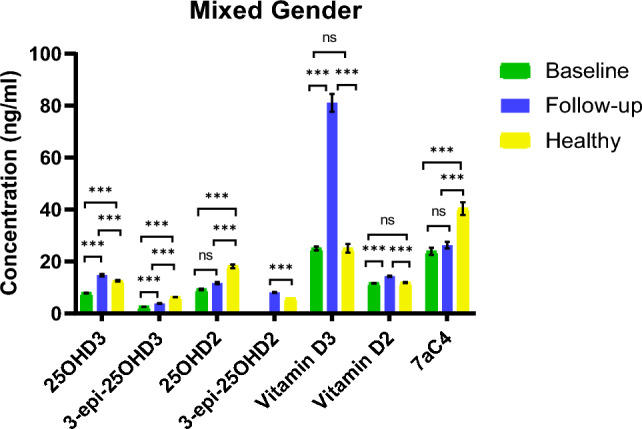
a comparison of the levels of vitamin D metabolites in plasma samples. In baseline subjects, 3-epi-25OHD2 was not detected, and in healthy, follow-up, and baseline subjects, 1α,25(OH)_2_D was not detected by the instrument. *p* < 0.05 = *, *p* < 0.01 = **, *p* < 0.001 = *** , ns = no significance.

In this study, the diagnostic criteria for vitamin D deficiency were less than 20 ng/ml (< 20 ng/ml) while the insufficiency ranged between 21 and 29 ng/ml and optimal were more than 30 ng/ml (> 30 ng/ml)^12^. The concentration of the active forms of vitamin D metabolites was increased when the baseline (obese subjects) took vitamin D supplements, as shown in Figs. 6, 7 and Table 5. The male and female obese subjects have high concentrations of vitamin D3 after vitamin D3 supplementation; consequently, there should be a significant increase of 25OHD3 and its epimer according to the fact that vitamin D3 undergoes hydroxylation process in the liver via CYP2R1 enzyme^41,42^. However, this study illustrated that although the 25OHD3 and its epimer increased significantly in supplemented subjects compared to baseline, the increase was insufficient to achieve the optimal range (> 30 ng/ml). A possible reason behind this is the possibility of vitamin D3 conversion to the other forms. For instance, vitamin D3 may be hydroxylated via the CYP11A1 enzyme and produce other forms of vitamin D, such as 22OHD3 and 20, 22(OH)_2_D3 in epidermal keratinocytes^19,43,44^. Furthermore, the CYP11A1 enzyme could be activated by UVB light (wavelength, 290–315 nm); as a result, the bioavailability of vitamin D3 substrate will be less for the generation of 25OHD3 and its epimer by classical hydroxylation^45^.

**Table 5:** Vitamin D metabolites concentrations in healthy, follow-up, and baseline of mixed-gender subjects. From left to right, the analyte’s name, the categories of the analysed samples, the number of samples (N), and the mean concentration in ng/ml with the standard deviation (SD).

No	Analyte	Sample type	N	Mean ± SD
1	Vitamin D3	Base line	277	25.03 ± 12.40
		Follow-up	277	81.09 ± 56.31
		Healthy	175	25.07 ± 21.96
2	Vitamin D2	Base line	277	11.68 ± 2.33
		Follow-up	277	14.34 ± 3.84
		Healthy	175	11.88 ± 2.79
3	25OHD3	Base line	277	7.89 ± 2.65
		Follow-up	277	14.76 ± 7.65
		Healthy	175	12.56 ± 3.84
4	3-epi-25OHD3	Base line	277	2.60 ± 0.48
		Follow-up	277	3.85 ± 2.10
		Healthy	175	6.26 ± 1.19
5	25OHD2	Base line	277	9.25 ± 4.52
		Follow-up	277	11.73 ± 6.94
		Healthy	175	18.18 ± 9.58
6	3-epi-25OHD2	Base line	277	n.d.*
		Follow-up	277	8.13 ± 2.94
		Healthy	175	5.88 ± 0.22
7	1α,25(OH)_2_D3	Base line	277	n.d.*
		Follow-up	277	n.d.*
		Healthy	175	n.d.*
8	1α,25(OH)_2_D2	Base line	277	n.d.*
		Follow-up	277	n.d.*
		Healthy	175	n.d.*
9	7αC4	Base line	277	23.88 ± 22.94
		Follow-up	277	26.27 ± 20.75
		Healthy	175	40.36 ± 31.84

*These metabolites were not detected by the UHPLC-MS/MS instrument.

The data from Figs. 6, 7 and Table 5 reveals that the levels of vitamin D2 and D3 are quite comparable between baseline and healthy control subjects. Moreover, it shows that vitamin D3 is extraordinarily high compared to 25OHD3. Let's delve into the reasons behind this:

Storage in Fat Cells: Vitamins D2 and D3 are fat-soluble. In obese individuals, these vitamins are stored in fat cells, or adipocytes. This means that, even if obese subjects have more of these vitamins in storage, it might not be readily reflected in their bloodstream.

Conversion to Active Form: The hydroxylation of vitamin D2 and D3 to their active form, 25OHD happens readily. This conversion is less efficient in obese subjects compared to healthy ones. One key reason for this is the lower expression of the CYP2R1 enzyme in obese individuals. As CYP2R1 facilitates the hydroxylation process, its reduced activity in obese patients means fewer vitamins D2 and D3 are transformed to 25OHD. Moreover, since the conversion of vitamin D3 to 25OHD3 is less efficient in obese subjects, the vitamin D3 concentration will be extraordinarily high compared to 25OHD3.

Effect on Vitamin D Levels: Despite obese individuals possibly having more stored vitamin D2 and D3 due to their fat content, the decreased conversion means that the bloodstream levels of these vitamins are quite similar to those in healthy individuals. However, because of this reduced conversion, the level of the active form, 25OHD, is actually higher in healthy individuals than in obese subjects.

Half-life Consideration: It's crucial to remember that vitamins D2 and D3 have a half-life of approximately 24 h, meaning they are metabolized relatively quickly. In contrast, their downstream metabolite, 25OHD, has a longer half-life of about 15 days. This longer duration combined with the inefficient conversion in obese subjects further accentuates the difference in 25OHD levels between the two groups.

Generally, while obese and healthy individuals might have similar bloodstream levels of vitamins D2 and D3, the reduced efficiency of conversion in obese subjects means they have lower levels of the active metabolite, 25OHD. This discrepancy underscores the importance of considering both storage and metabolic factors when assessing vitamin D status^46^.

According to Table 5, when 25OHD2, 3-epi-25OHD2, 25OHD3, 3-epi-25OHD3 are summed together as total 25OHD, the follow-up and healthy subjects will have sufficient 25OHD (> 30 ng/ml). The immunoassay technique measures the total 25OHD, which overestimates the actual 25OHD3 and gives false-positive results. This means that the UHPLC-MS/MS technique is accurate, precises, and specific since it can distinguish between vitamin D metabolites.

A total of 277 participants, with an average age of 41 ± 12 years, were part of a 6-month study, of which 204 (74%) were female. Based on the WHO BMI classification, 65 participants (25%) had a normal BMI, 92 (35%) were overweight, and 106 (40%) were classified as obese at the beginning of the study. Table 6 presents the initial clinical, metabolic, inflammatory and lipid profile markers for the overweight and obese groups in comparison to the normal weight group. From the 277 participants, there was a noticeably higher prevalence of diabetes and hypertension in the overweight and obese categories than in the normal BMI category, as indicated in Table 6. Both at the start and end of the study, blood pressure, inflammatory markers, metabolic markers, and lipid profiles, specifically LDL & TG, were observably elevated, while HDL showed a marked decrease in the overweight and obese groups compared to the normal weight group, as shown in Table 6. Detailed information regarding the correlations between vitamin D metabolites and the severity of weight excess (BMI) and C-reactive protein (CRP) is provided in the electronic supplementary material (ESM).

**Table 6:** Average clinical and metabolic risk factors at baseline, based on BMI cut-off values [mean and standard deviation (SD) in brackets, *p* value ≤ 0.05].

Patient data	Normal (BMI = 18.5–24.9) (n = 65)	Overweight (BMI = 25–29.9) (n = 92)	Obese (BMI = ≥ 30) (n = 106)	*p* value
Age (years)	34 (12)	43 (10)	45 (11)	0
Male:female n(%)	52 (80)	62 (67)	80 (74)	0.2
Smoking n(%)	5 (8)	15 (16)	15 (14)	0.9
Diabetes n(%)	6 (9)	18 (19)	21 (19)	0.2
Hypertension n(%)	3 (5)	14 (15)	24 (22)	0.0
Ischaemic heart disease n(%)	1 (2)	1 (1)	4 (4)	0.4
Systolic BP (mmHg)	117 (9)	126 (18)	126 (15)	0.001
Diastolic BP (mmHg)	73 (7)	76 (7)	78 (8)	0.01
hs-CRP (mg/L)	2.25 (2.8)	3.4 (3.8)	4.5 (4)	0
Glucose (mmol/L)	5.4 (1.7)	6.4 (2.8)	6.9 (3.1)	0
HbA1c (%)	5.42 (0.5)	5.86 (1.1)	6.08 (1.1)	0
Total cholesterol(mmol/L)	4.64 (1.1)	4.96 (0.9)	4.93 (0.9)	0.088
Triglycerides (mmol/L)	1.09 (0.6)	1.69 (1.2)	1.67 (1.2)	0.001
LDL (mmol/L)	3.07 (0.9)	3.34 (0.86)	3.39 (0.86)	0.055
HDL (mmol/L)	1.39 (0.43)	1.26 (0.37)	1.13 (0.36)	0

**Figure 8:**
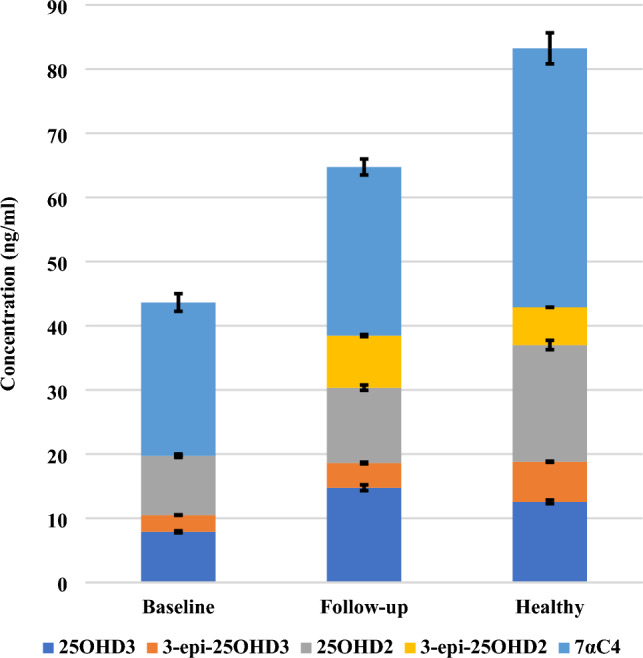
A comparison between the concentration of vitamin D metabolites in mixed gender of healthy, follow-up, and baseline subjects. The stacked column in the chart illustrates the sum of 25OHD and its epimer and isobar when these metabolites are not separated sufficiently. The Error bars describe the standard errors of the mean. .

**Figure 9:**
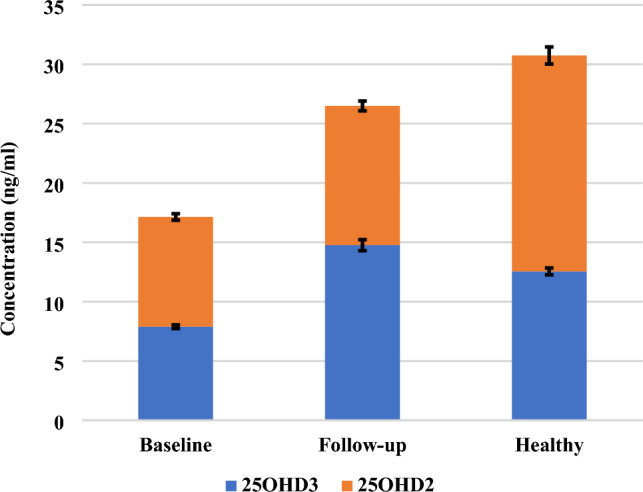
A comparison between the concentration of 25OHD2 and 25OHD3 after the epimers and isobar were excluded in mixed gender of healthy, follow-up, and baseline subjects. The stacked column in the chart illustrates the sum of 25OHD2 and 25OHD3 when these two metabolites are not distinguished from each other adequately. The Error bars describe the standard errors of the mean.

When the epimer and isobar are not separated adequately from 25OHD3, they will interfere and overestimate the actual levels of 25OHD3, as shown in Fig. 8. The baseline, follow-up, and healthy subjects are considered to have deficient levels of 25OHD3 and 25OHD2 when epimers and isobars are removed from the commonly measured 25OHD, as shown in Fig. 9. Also, when 25OHD2 and 25OHD3 are summed together, the healthy subjects will have optimal levels of 25OHD (> 30 ng/ml), the follow-up subjects will be in the insufficient range (21–29 ng/ml), and the baseline subjects will have deficient levels of 25OHD (< 20 ng/ml), as shown in Fig. 9. Typically, this study demonstrates the power of the UHPLC-MS/MS because this technique excludes the epimer and isobar from 25OHD by separating them based on their polarity, retention time, fragmentation behavior, and mass-to-charge ratio (m/z) leading to accurate results. Finally, the comparison of the data obtained by the immunoassay/commercial chemiluminescence vitamin D assay and those obtained by the UHPLC-MS/MS assay was published previously^47^.

## Materials and methods

### Standards and chemicals

25-hydroxyvitamin D3 (6, 19, 19-d3), 1-α-25-dihydroxyvitamin-D2 (1α,25(OH)_2_D2), 1-α-25 dihydroxyvitamin-D3 (1α,25(OH)_2_D3), 25-hydroxyvitamin-D2 (25OHD2), 3-epi-25- hydroxyvitamin-D2 (3-epi-25OHD2), 25-hydroxyvitamin-D3 (25OHD3), 3-epi-25- hydroxyvitamin-D3 (3-epi-25OHD3), vitamin-D2, vitamin-D3, 7- α-hydroxy-4-cholesten-3-one (7αC4), phosphate buffered saline, albumin from human serum, formic acid and ammonium formate for LC–MS were purchased from Sigma-Aldrich. LC–MS-grade methanol and LC–MS-grade water were purchased from Merck [United States]. Hexane and ethyl acetate were purchased from Fisher Scientific [Hampton, New Hampshire, United States].

### Preparation of standards solution

The stock solution (1 mg/ml) of each vitamin D metabolite was prepared by dissolving 1 mg of powder form of each vitamin D metabolite in 1 ml of methanol and stored at – 80 ℃. Since the vitamin D metabolites are light-sensitive, the preparation of stock solutions should be in the dark and maintained in amber vials. The working standard solutions or the desired concentration were formulated in methanol by serial diluting the stock solution.

### Collection of blood samples

Blood samples were taken from 452 subjects, 277 baselines (obese subjects), 277 follow-ups (supplemented obese subjects), and 175 healthy subjects. These individuals (baseline and follow-ups) were 73 males (age range 18–82) and 204 females (age range 18–65), while healthy volunteers were 8 males (age range 18–29) and 167 females (age range 18–65). Blood samples were centrifuged at 2500 × *g* for 10 min to extract the serum from the blood; the serum was maintained in amber tubes at – 80 ℃ until analysis. The College of Medicine in the United Arab Emirates University approved this recent vitamin D study with the ethics approval number (AAHEC-3–17-055), and all participants provided their consent. Our research primarily targeted Emirati citizens and Middle Eastern expatriates participating in a randomized, double-blind, placebo-controlled trial involving vitamin D3 supplementation (1000 IU/day), optionally combined with calcium. Participants over the age of 18 were recruited using various mediums such as local press advertisements, community health centers, and hospital outpatient clinics. Individuals with conditions such as renal disease or hypercalcaemia or those using supplements or specific medications were excluded.

After providing their informed consent, we collected blood samples from the participants to measure vitamin D metabolites and to screen for inflammatory and metabolic risk markers. Additionally, we collected baseline data and information on several variables that could potentially influence vitamin D status, including lifestyle and health-related factors, demographic details, dietary habits, and physical activity levels. All this information was collected through a detailed face-to-face questionnaire. We used a Tanita body composition analyser to measure participants' anthropometric data such as body weight, height, and BMI. According to WHO's sex-adjusted BMI cut-off points, we classified participants as normal (BMI 18.5–24.9), overweight (BMI 25–29.9), and obese (BMI 30 or above).

### Plasma samples extraction method

The previously frozen serum samples from humans were thawed at room temperature for around 20 min. Then, they were vortexed. The sample volume used to initiate liquid–liquid extraction was 500 μl. After that, 20 ul of internal standard (1 ug/ml) was added to all samples, quality controls, and calibrants except the blank. A mixture of 1.0 ml of ethyl acetate: hexane (1:9) was used as the extraction solvent to extract vitamin D metabolites from serum. After that, vortexing was applied to the mixture for a few seconds; then, the centrifugation was performed at room temperature at 4000 × *g* for 20 min. The supernatant layer was extracted and transferred into a new tube; further extraction was applied to the remaining lower layer. All the extract supernatant layers were pooled together into a new glass tube. Afterward, a gentle stream of nitrogen gas was applied to dry the extracts at room temperature using a sample concentrator. Finally, the residue was resuspended by adding 100 μl of water: methanol (25:75, v/v) mixture LC–MS/MS grade for the analysis by UHPLC-MS/MS.

### Instrumentation

The UHPLC-MS/MS instrument consists of a Nexera ultra high-performance liquid chromatography (UHPLC) coupled with a triple quadrupole mass spectrometer (Shimadzu, model 8060, Japan). The UHPLC system comprises a degasser, column oven, auto-sampler, and pump. It employs a column with a small particle size and diameter since the UHPLC system can tolerate high pressure. Also, it is characterized by utilizing multiple solvents simultaneously and a high injection speed. The tandem mass spectrometer system includes a triple quadrupole with an innovative UF-Qarray ion guide. This ion guide enhances the sensitivity of the UHPLC-MS/MS by amplifying the intensity of the ion signal and minimizing the noise background level. The UHPLC-MS/MS-8060 from Shimadzu company provides excellent responsiveness, speed, sensitivity, and selectivity.

**Figure 10:**
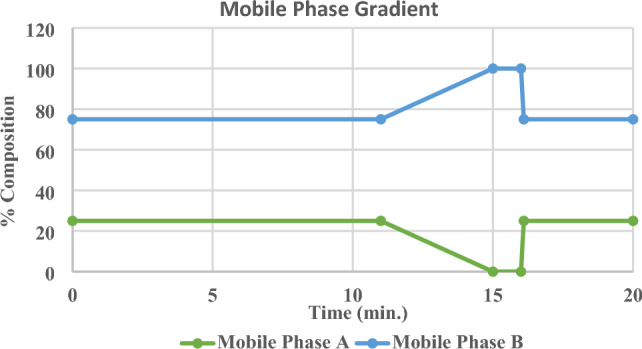
Mobile phase gradient elution profile. Mobile phase A consisted of 5 mM ammonium formate and 0.1% formic acid in LC–MS grade water. Mobile phase B consisted of 5 mM ammonium formate and 0.1% formic acid in LC–MS grade methanol. The mobile phase composition is changing over time. For example, from 0:00 to 11:00 min., mobile phase A and B were maintained at 25% and 75%, respectively. After that, mobile phase B was increased to 100% at 15 min. which kept for one min followed by an increase in mobile phase A to 25% at 16:10 min and kept until 20:00 min. for equilibration step.

The reversed-phase analytical column (Ascentis Express F5 column, dimensions: 2.7 µm × 2.1 mm × 150 mm) was used to separate vitamin D metabolites. The guard column was linked to the analytical column for physical filtration and extending the lifespan of the analytical column. 5 ul of the sample volume was injected into the system. After every injection, a wash program was employed to wash the needle with a methanol/water (75:25, v/v) to reduce carryover and contamination. The mobile phase flow rate was adjusted to 0.5 ml/min while the column’s temperature was maintained at 30 ℃. Mobile phase A (LC–MS grade water + 0.1% formic acid + 5 mM ammonium formate) and Mobile phase B (LC–MS grade methanol + 0.1% formic acid + 5 mM ammonium formate) were employed for chromatographic separation utilizing a binary mobile phase gradient pump mode. The optimum mobile phase gradient for separation vitamin D metabolites is shown in Fig. 10.

In the tandem-mass spectrometry system, a positive electrospray ionization (ESI) mode was applied to all vitamin D metabolites. The nebulizing, drying, and heating gas flow were set at 2, 8, 8 L/min, respectively. Interface and heating block temperatures were maintained at 300 and 400 ℃, respectively. The UHPLC-MS/MS parameters were controlled by using Shimadzu’s Lab Solutions software.

### Method development and validation

Any bioanalytical method should be developed and validated for linearity of calibration curve, precision, accuracy, sensitivity, specificity, stability, and recovery according to U.S. Food and Drug Administration (FDA)^48,49^. The calibrants for calibration curve and three levels of QCs (QCL, QCM, QCH) were prepared in a surrogate matrix containing a mixture of phosphate-buffered saline with albumin from human serum (artificial serum) at a concentration of 60 g/L. After that, the QCs were analysed by the instrument and the data was used to validate the method.

### Statiscal analysis

The statistical analyses were performed using the GraphPad Prism software (version 8.01, Inc., 2012, USA) and SPSS software (version 25, SPSS Inc., Chicago). The statistical analysis was carried out by one-way ANOVA followed by Newman-Keuls multiple comparisons test to find the significant difference between the three groups (Baseline, Follow-up, Healthy). Unpaired t test was used to test the significant differences between two groups; Follow-up vs the Healthy group of 3-epi-25OHD2. Data are shown as mean ± S.E.M. in graphs and as mean ± SD in tables. Multiple regression analysis was also used to determine the independent influence of BMI on metabolic, inflammatory and lipid profile risk factors after adjusting for important prognostic factors including age, gender, smoking and type 2 diabetes and a *p*- value < 0.05 was considered significant.

## Institutional review board and informed consent

The study was conducted in accordance with the Declaration of Helsinki, and approved by the Institutional Review Board (or Ethics Committee) of Al Ain Medical District Human Research Ethics Committee approved the study protocol and consent to participate form (Reference number: AAMDHREC protocol no 14/15). Written consent was obtained from all patients recruited for this study. Informed consent was obtained from all subjects involved in this study.

## Conclusions

In this study, the method for the vitamin D metabolites analysis in human blood samples was optimized, developed and validated to qualify and quantify these metabolites precisely and accurately. The universal measured form of vitamin D (25OHD) should be separated and distinguished from its epimer and isobar to avoid overestimating its measurements and get accurate results. The results from this study showed that when basline obese subjects were supplemented with vitamin D3, the vitamin D3 and its active form (25OHD3) levels were increased significantly; however, the 25OHD3 increase was insufficient to achieve the normal range. Moreover, the baseline and healthy subjects have comparable levels of vitamin D3 and D2; furthermore, vitamin D3 concentration was extremely high compared to 25OHD3 for some possible factors which include storage in fat cells and the efficiency of conversion to the active form. This study highlights the importance of considering storage and metabolic effects when assessing vitamin D status in the subjects. Lastly, the method used to analyse vitamin D metabolites in the blood could be helpful for application in several vitamin D-related studies.

### Supplementary Information

Supplementary Information.

## Data Availability

All data generated or analysed during this study are included in this published article and its supplementary information files. **Sample availability:** Samples of the compounds are available from the authors.

## Abbreviations

1α,25(OH)_2_D2: 1-α-25 Dihydroxyvitamin-D2
1α,25(OH)_2_D3: 1-α-25 Dihydroxyvitamin-D3
25OHD2: 25-Hydroxyvitamin-D2
25OHD3: 25-Hydroxyvitamin-D3
3-epi-25OHD2: 3-Epi-25-hydroxyvitamin-D2
3-epi-25OHD3: 3-Epi-25-hydroxyvitamin-D3
7αC4: 7-α-Hydroxy-4-cholesten-3-one
22(OH)D3: 22-Hydroxyvitamin-D3
20,22(OH)_2_D3: 20,22-Dihydroxyvitamin-D3
7DHC: 7-Dehydrocholesterol
7DHP: 7-Dehydropregnenolone
22(OH)7DHC: 22-Hydroxy-7-dehydrocholesterol
20,22(OH)_2_7DHC: 20,22-Dihydroxy-7-dehydrocholesterol
20(OH)D3: 20-Hydroxyvitamin-D3
17,20,23(OH)_3_D3: 17,20,23-Trihydroxyvitamin-D3
BMI: Body mass index
BP: Blood pressure
CYP27B1: Cytochrome p450 27B1
CYP2R1: Cytochrome P450 2R1
CYP11A1: Cytochrome P450 11A1
CV: Coefficient of variation
DBP: Vitamin D-binding protein
ESI: Electrospray ionization
FDA: Food and drug administration
HPLC: High performance liquid chromatography
HS-CRP: High-sensitivity C-reactive protein
HbA1c: Hemoglobin A1c
HDL: High-density lipoprotein
LDL: Low-density lipoprotein
LC–MS/MS: Liquid chromatography-tandem mass spectrometry
LOD: Limit of detection
LOQ: Limit of quantitation
MRM: Multiple reaction monitoring
N: Number
PE: Error percentage
QC: Quality control
QCs: Quality controls
QCH: Quality control high
QCL: Quality control low
QCM: Quality control medium
R^2^: Coefficient of determination
S/N: Signal to noise ratio
SD: Standard deviation
S.E.M: Standard error of the mean
TG: Triglycerides
UHPLC-MS/MS: Ultra-high-performance liquid chromatography–tandem mass spectrometry
UV-B: Ultraviolet-B
WHO: World health organization
