# Supplementary material for: Unraveling the complex interplay between obesity and vitamin D metabolism
Source: Sci Rep. 2024 Mar 30;14:7583. doi: 10.1038/s41598-024-58154-z (PMC10981658; doi:10.1038/s41598-024-58154-z)
Supplement: Supplementary file 1 — Supplementary Information. [file 41598_2024_58154_MOESM1_ESM.docx]

**ELECTRONIC SUPPLEMENTARY MATERIAL**

Unravelling the Complex Interplay among Vitamin D Metabolites in Obesity: Insights from an LC-MS/MS Study

**Supplementary Table and Figure given below**

Table S1. Correlations between vitamin D metabolites and the severity of weight excess (BMI) and CRP

| **Vitamin D Metabolite** | **BMI** | **CRP** |
| --- | --- | --- |
| Vitamin D3(ng/ml) | r=.004  p=0.948 | r=-.081  p=0.181 |
| Vitamin D2(ng/ml) | r=.001  p=989 | r=-.055  p=0.359 |
| 25OHD3(ng/ml) | r=.040  p=0.513 | r=-.073  p=0.227 |
| 25OHD2(ng/ml) | r=.234  p=0.349 | r=.164  p=0.490 |
| 1α,25(OH)_2_D3 (ng/ml) | r=.221  p=0.001 | r=.088  p=0.203 |
| 3-Epi-25OHD3 (ng/ml) | r=.169  p=0.025 | r=.007  P=0.930 |
| 7αC4 (ng/ml) | r=.141  p=0.021 | r=.017  P=0.779 |

Abbreviations: 25OHD3, 25-hydroxyvitamin-D3; 25OHD2, 25-hydroxyvitamin-D2; 1α,25(OH)_2_D3, 1-α-25 dihydroxyvitamin-D3; 3-Epi-25OHD3, 3-epi-25-hydroxyvitamin-D3; 7αC4, 7-α-hydroxy-4-cholesten-3-one; BMI, Body mass index; CRP, C-reactive protein.


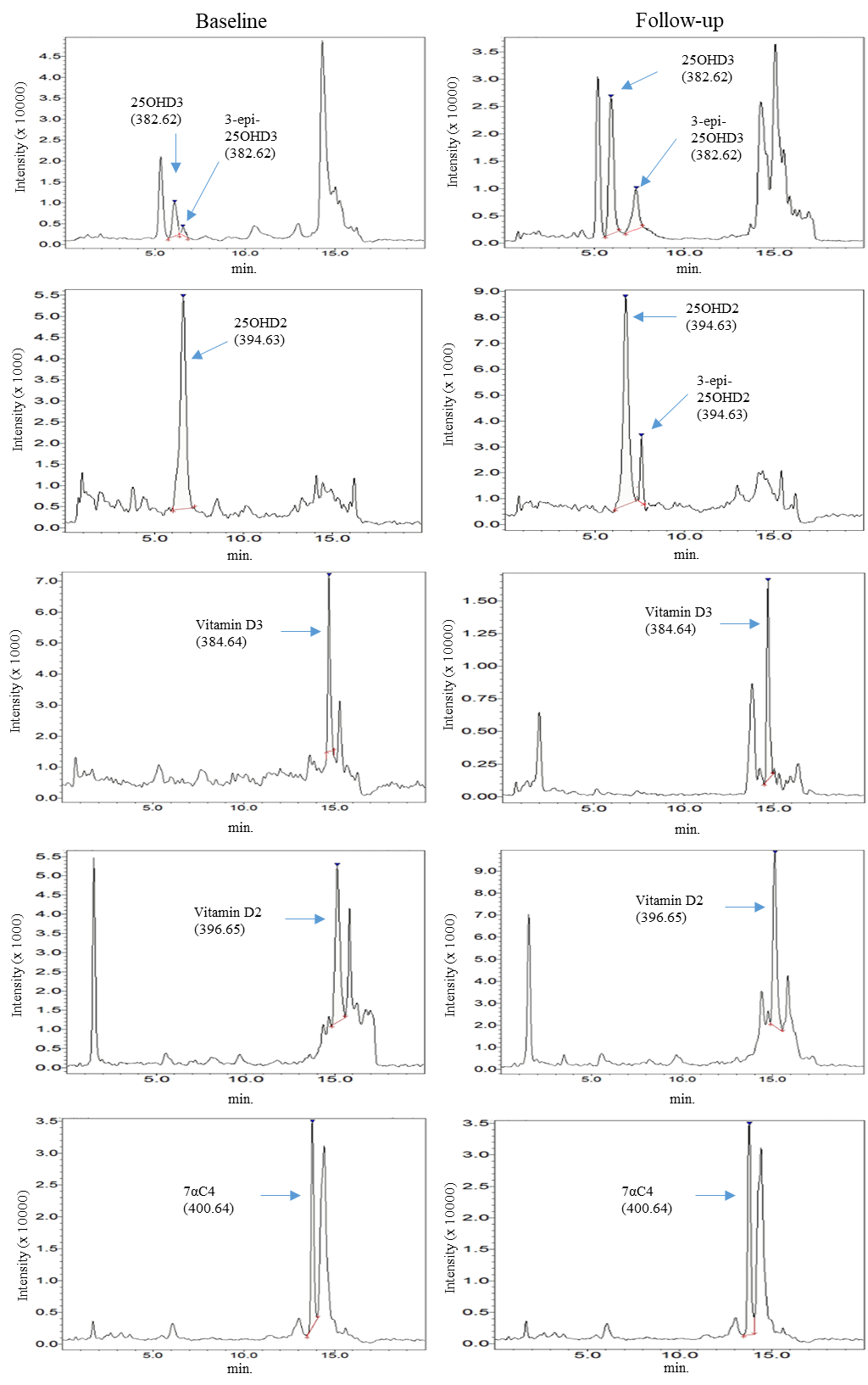


Figure S1. Vitamin D metabolites Chromatogram in human samples. The chromatogram illustrates the vitamin D metabolites along with their molecular weights expressed in g/mol in two obese groups (Baseline and Follow-up). 3-epi-25OHD2 was not detected in baseline group.
